# Delivery of shRNA via lentivirus in human pseudoislets provides a model to test dynamic regulation of insulin secretion and gene function in human islets

**DOI:** 10.14814/phy2.13907

**Published:** 2018-10-28

**Authors:** Mikako Harata, Siming Liu, Joseph A. Promes, Anthony J. Burand, James A. Ankrum, Yumi Imai

**Affiliations:** ^1^ Department of Internal Medicine Carver College of Medicine University of Iowa Iowa City Iowa; ^2^ Fraternal Order of Eagles Diabetes Research Center University of Iowa Iowa City Iowa; ^3^ Department of Biomedical Engineering University of Iowa Iowa City Iowa

**Keywords:** Beta cell, diabetes, extracellular matrix, glucokinase, inflammation

## Abstract

Rodent islets are widely used to study the pathophysiology of beta cells and islet function, however, structural and functional differences exist between human and rodent islets, highlighting the need for human islet studies. Human islets are highly variable, deteriorate during culture, and are difficult to genetically modify, making mechanistic studies difficult to conduct and reproduce. To overcome these limitations, we tested whether pseudoislets, created by dissociation and reaggregation of islet cell suspensions, allow for assessment of dynamic islet function after genetic modulation. Characterization of pseudoislets cultured for 1 week revealed better preservation of first‐phase glucose‐stimulated insulin secretion (GSIS) compared with cultured‐intact islets and insulin secretion profiles similar to fresh islets when challenged by glibenclamide and KCl. qPCR indicated that pseudoislets are similar to the original islets for the expression of markers for cell types, beta cell function, and cellular stress with the exception of reduced proinflammatory cytokine genes (*IL1B, CCL2, CXCL8*). The expression of extracellular matrix markers (*ASPN, COL1A1, COL4A1*) was also altered in pseudoislets compared with intact islets. Compared with intact islets transduced by adenovirus, pseudoislets transduced by lentivirus showed uniform transduction and better first‐phase GSIS. Lastly, the lentiviral‐mediated delivery of short hairpin RNA targeting glucokinase (*GCK*) achieved significant reduction of *GCK* expression in pseudoislets as well as marked reduction of both first and second phase GSIS without affecting the insulin secretion in response to KCl. Thus, pseudoislets are a tool that enables efficient genetic modulation of human islet cells while preserving insulin secretion.

## Introduction

The loss of functional beta cell mass is the central pathology for both type 1 and type 2 diabetes (Kahn 2001; Atkinson et al. 2014; Chen et al. 2017). As the three‐dimensional structure of pancreatic islets supports viability and function of beta cells through cell‐cell and cell‐matrix communications (Rutter and Hodson 2015; Arous and Wehrle‐Haller 2017; Reissaus and Piston 2017; Briant et al. 2018), it is critical to address beta cell pathophysiology in pancreatic islets. Rodent islets are readily available, cost effective, can be easily genetically manipulated, and can be compared with syngeneic animals to connect in vitro observations to in vivo phenotype. However, human islets differ substantially from their rodent counterparts anatomically and functionally. Humans and mice show distinct islet innervation, cell distribution, and ratio of beta to alpha cells (Arrojo e Drigo et al. 2015). Glucose transporters, ion channels, the ratio of first/second phase of glucose‐stimulated insulin secretion (GSIS), and amyloid deposition also differ between human and mouse islets (Arrojo e Drigo et al. 2015; Dai et al. 2016; Skelin Klemen et al. 2017). Thus, studies of human islets from organ donors are important for understanding the regulation of islet function and beta cell viability in humans.

However, human islets are limited in availability, costly, difficult to maintain in culture, and challenging to genetically modify. Islet function, including GSIS, reduces over time in culture (Paraskevas et al. 2000; Arzouni et al. 2018). Genetic manipulation of intact islets by liposomal or viral‐mediated vehicles has low efficiency and typically requires partial dispersion or enzyme digestion that compromises insulin secretion and removes cell‐cell communication. The enormous heterogeneity of islet sizes also introduces high variability in assays. To overcome the variability of isolated human islets, islet spheroids or pseudoislets composed of dissociated reaggregated islet cells has been used. Reaggregated islet cells form uniformly sized pseudoislets that maintain similar spatial distribution of beta and alpha cells with better first phase GSIS compared with dispersed cells (Hopcroft et al. 1985; Halban et al. 1987) and original islets (Zuellig et al. 2017; Yu et al. 2018). Pseudoislets also lend themselves to more efficient gene modification (Caton et al. 2003; Arda et al. 2016; Peiris et al. 2018). Caton et al. reported that lentiviral‐mediated overexpression of connexin cDNA does not interfere with pseudoislet formation and allows for the transduction of a large proportion of cells (Caton et al. 2003). Thus, pseudoislets appear to offer a unique and useful model to assess human islet function after the modulation of gene expression. Using readily available reagents and resources, we first characterized pseudoislets for insulin secretion in response to secretagogues by perifusion and analyzed their expression of markers for islet cell types, beta cell function, cell stress, and extracellular matrix (ECM). We compared transduction efficiency between adenovirus and lentivirus side by side and determine the impact of transduction on GSIS by perifusion. Following characterization of the pseudoislet platform, we tested short hairpin RNA (shRNA) delivered with a lentiviral vector targeting glucokinase (GCK) as a model target to test dynamism of insulin secretion by perifusion. Our data demonstrate that lentiviral‐mediated gene downregulation combined with a simple protocol to form human pseudoislets is a useful tool that enables assessment of the impact of gene function on islet GSIS.

## Methods

### Human islets

**Figure 1:**
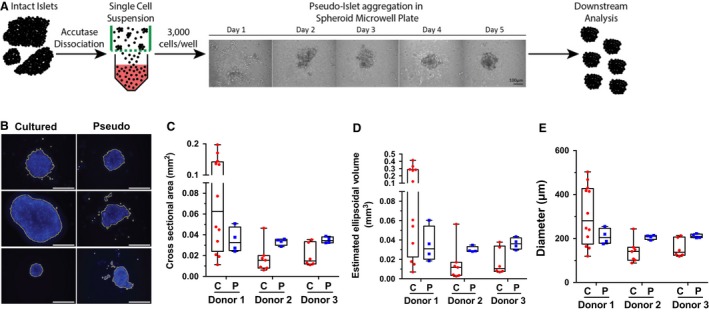
Pseudoislets exhibit a narrower size distribution compared with cultured‐intact islets. (A) Pseudoislet formation process from fresh islets using spheroid microwell plates. (B) Representative images of pseudo or cultured‐intact islets from donor 1 stained with nuclear dye Hoechst 33342 analyzed by ImageJ macro. Analysis marks shown in yellow. Scale bar = 250 *μ*m. (C) Quantified cross‐sectional area from cultured‐intact C and pseudoislets (P) using max‐projected fluorescent images analyzed by ImageJ macro. (D) Estimation of ellipsoidal volume of culture‐intact or pseudoislets based on major and minor axis measurements extracted from fluorescent images by ImageJ macro. (E) Diameter of cultured‐intact or pseudoislets obtained as in methods. Mean ± SEM, *n* = 12 (donor 1, culture‐intact), 7 (donor 2, culture‐intact), 6 (donor 3, culture‐intact), 4 (all donors, pseudoislets).

Human islets from nondiabetic donors from Integrated Islet Distribution Program or PRODO laboratories (Table 1) with reported viability and purity above 80% were cultured in CMRL1066 containing 1% human serum albumin (HSA), 1% Pen‐Strep, and 1% l‐Glutamate (1% HSA CMRL) overnight at 37°C and 5% CO_2_ upon arrival for recovery from shipping. Then, islets were divided into fresh, cultured‐intact, or pseudoislets. While fresh islets were harvested on the next day, cultured‐intact islets were maintained in CMRL1066 containing 10% heat inactivated FBS, 1% Pen‐Strep, and 1% l‐Glutamate (10% HI‐FBS CMRL) for 1 week at 37°C and 5% CO_2_ before harvesting. For pseudoislets preparation (Fig. 1A), single cell suspension was prepared first as follows. Human islets were washed once with PBS, digested with Accutase (A6964, MilliporeSigma, St Louis, MO) at 37°C for 5 min, pipetted through 1 mL tip for 15 times, digested for additional 4 min at 37°C, and passed through 40 *μ*m strainer using a plunger of 1 mL syringe (Butcher et al. 2014). Filtered single cell suspension was counted, washed with PBS once, resuspended in 10% HI‐FBS CMRL at 10^2^ cells/*μ*L, and seeded in a 96‐well spheroid microwell plate (Corning, Corning, NY) at 3000 cells/well. The microwell plate was centrifuged at 270*g* at room temperature for 7 min and cultured after addition of 100 *μ*L/well of 10% HI‐FBS CMRL at 37°C and 5% CO_2_ until analyses. The study was reviewed by IRB at University of Iowa and approved as nonhuman study.

**Table 1:** Characteristics of islet donors

Donor	Sex	Age (years)	BMI (kg/m^2^)	Race^a^	Cause of death	Study done^b^	Source
1	F	42	32.10	Wh	Cerebrovascular/stroke	Figures 1B–D, 2A–C and 5A–C	IIDP
2	M	45	29.3	Bl	Cerebrovascular/Stroke	Figures 1C , D, 3 and 4C , D	IIDP
3	M	62	36.1	Bl	Anoxia	Figure 1C and D	IIDP
4	M	45	24.70	Hisp	Subdural Hygroma	Figure. 3 (except for SST, GHRL, ACTA2, AMY2A); Figure 4A–D	IIDP
5	M	48	32.4	Hisp	Cerebrovascular/Stroke	Figures 3 and 4A–D	Prodo
6	F	59	36.1	Hisp	Anoxia	Figures 3, 4A–C	Prodo
7	M	32	32.30	Wh	Anoxia	Figures 2B, C, 3, 4A–D and 5C–G	IIDP
8	M	58	31.8	Wh	Cerebrovascular/Stroke	Figures 2B, C, 3, and 4A–D, 5C, D, F, G	Prodo
9	F	47	24.1	Wh	Cerebrovascular/Stroke	Figures 2B,C, 3 and 5C, D, F, G	IIDP
10	F	57	25.80	Wh	Cerebrovascular/Stroke	Figure 4A	IIDP
11	M	53	26	Wh	Head Trauma	Figures 2A–D, 4A, B	Prodo
12	M	64	34.5	Wh	Head Trauma	Figures 2A–C, 4A, B, and 5D	IIDP
13	M	49	34	Hisp	Cerebrovascular/Stroke	Figures 4B and 5D	IIDP
14	M	56	40.60	Wh	Head Trauma	Figure 2E–H	IIDP
15	M	30	25.9	Wh	Anoxia	Figure 2E–H	IIDP
16	M	27	30	Wh	Head Trauma	Figures 2A–C, 4A–D	IIDP
17	M	38	33	Wh	Head Trauma	Figure 2A	IIDP
18	M	55	28.5	Wh	Anoxia	Figure 2A–C	IIDP
19	F	57	25.8	Wh	Cerebrovascular/stroke	Figure 2A–C	IIDP
20	M	55	30.1	Wh	Head trauma	Figure 2E–H	IIDP
21	M	51	29	Wh	Head trauma	Figure 2E–H	IIDP

a Hisp; Hispanic, Wh; White, Bl; Black.

b Studies were assigned based on availability of number of islets and an order of islets being received but not on donor characteristics

### ImageJ analysis of islet size

Islets in 10% HI‐FBS CMRL were incubated with 10 *μ*g/mL Hoechst 33342 for 30 min at 37°C and 5% CO_2_. Then, Z‐stack images captured by Leica DMi8 Microscope (Leica Microsystem, Buffalo Grove, IL) were analyzed with the ImageJ macro “Measure Spheroid Shape.ijm.”(https://www.researchgate.net/publication/326844033_ImageJ_Macro_to_Quantify_Spheroid_Volume_and_Size). The maximal intensity Z‐stack was converted into a single plane and a binary threshold was applied to create a mask surrounding the islet cell mass. A series of erosions and dilations were performed to remove debris and the ImageJ plugin “Measure Particles” was used to find the dimensions of the islet fit using an ellipse approximation as well as directly measure the “Area” of the max projected islet cross section. The major and minor diameters of the calculated ellipse were then used to estimate the ellipsoidal volume of the islet that was defined as *V* = (4/3)*π*a*b^2^, where (a) is the major ellipse axis and (b) is the minor ellipse axis. Diameter was calculated as 0.5*square root of (area/*π*). The macro was run with the restrictions of circularity between 0.5 and 1 and islet area <18% of the total image area. Images which did not pass these quality control metrics were flagged, reviewed, and the image threshold was set manually.

### Perifusion of islets

BioRep Perifusion System (Biorep Technologies, Miami Lakes, FL) was used to perifuse human islets at 120 *μ*L/min and perfusates were collected every minute between 49 to 58 min and every 2 min for the rest of the run. After 52 min in 2.8 mmol/L glucose in Krebs‐Ringer bicarbonate (KRB) buffer, islets were perifused for 16 min with 16.7 mmol/L glucose, 10 *μ*mol/L glibenclamide in 5.6 mmol/L glucose, or 30 mmol/L KCl in 2.8 mmol/L glucose followed by 2.8 mmol/L glucose in vivo ose alone in KRB unless specified otherwise. Total insulin contents were obtained from islets incubated overnight at 4°C in acidified ethanol. Insulin was measured using STELLUX Chemiluminescent Human Insulin ELISA (ALPCO, Salem, NH). Insulin secretion was expressed by taking total insulin contents as 100% (% total) when the comparison is made among islets from the same donor or taking the average of insulin secretion during perifusion at 2.8 mmol/L (basal) as 1 when data from multiple donors were combined due to large variation of % total among donors and the lack of correlation between % total and efficiency of GSIS (Butcher et al. 2014). Stimulation index (SI) for the first phase was determined as the average insulin secretion between 53 and 56 min and SI for the second phase as the average of insulin secretion between 57 and 70 min, both divided by average basal insulin secretion.

### mRNA and quantitative PCR

RNA was isolated from islets using TRIzol reagent (ThermoFisher Scientific, Waltham, MA) according to manufacturer's protocol and cDNA was synthesized using Superscript IV VILO Master Mix (ThermoFisher Scientific). Gene expressions were assessed using ABI TaqMan commercial primers (Applied Biosystems, Foster City, CA) and results were expressed taking human PPIB as an internal standard.

### Lentivirus production and transduction of islets

Lentiviral vector expressing GFP under CMV promoter (LV‐CMV‐GFP) was obtained from University of Iowa Viral Vector Core (Iowa city, IA). Scramble and ShRNA sequence targeting human GCK obtained from Genetic Perturbation Platform (https://portals.broadinstitute.org/gpp/public) were cloned into PLKO.1 vector (10878, Addgene, Cambridge, MA) under human U6 promoter. Seventy percent confluent HEK293T cells in a 10 cm plate were transfected with 15 *μ*g PLKO1‐shRNA, 9 *μ*g pMDLg/pRRE (12251, Addgene), 5.5 *μ*g pRSV‐Rev (12253, Addgene), and 5 *μ*g pMD2.G (12259, Addgene) mixed with 103.5 *μ*g PEI (MilliporeSigma) for 6 h and grown for 60 h in 10% FBS DMEM with 1% Pen‐Strep, and 1% l‐Glutamate. Thereafter, virus‐containing media were centrifuged at 250*g* and filtered through a 0.45 *μ*m filter. Viruses were pelleted by ultracentrifugation at 125,000*g* for 2 h, resuspended in 100 *μ*L PBS, and stored in −80°C.

For pseudoislets transduction, islet single cell suspension in 10% HI‐FBS CMRL prepared as above was mixed with lentiviruses at approximately 0.8 x 10^6^ TU/3000 islet cells and seeded in 96‐well spheroid microwell plates at 30 *μ*L/well as above except that 100 *μ*L 10% HI‐FBS CMRL was added after the overnight culture at 37°C and 5% CO_2_. For cultured‐intact islet transduction, intact islets after overnight culture were resuspended in 0.1 mmol/L EGTA in serum free CRML and incubated with adenovirus expressing GFP under CMV promoter (Ad‐CMV‐GFP from Vector Biolabs, Malvern, PA) at 10,000 pfu/IEQ for 1 h at room temperature with mixing every 15 min before transferring to 10% HI‐FBS CRML for culture.

### Statistics

Data are presented as mean ± SEM or SD as specified. Differences of numeric parameters between two groups were assessed with Student's *t*‐tests. Paired test was used when all values are paired between groups. Welch correction was applied when variances between two groups were significantly different by *F* test using Prism 7 (Graphpad, La Jolla, CA). A *P* < 0.05 was considered significant.

## Results

### Pseudoislets maintain first‐phase insulin secretion better than cultured intact islets

As one of the primary advantages of pseudoislets is their relatively uniform size compared to native islets (Yu et al. 2018), we first assessed the variability of pseudoislet size compared with native islets for three human donors. Cross‐sectional area of pseudoislets and intact islets was measured and the ellipsoidal volume was estimated from Z‐stack images of Hoechst‐stained samples (Fig. 1B). Cultured‐intact islets showed substantial variability in cross‐sectional area and volume within each donor as well as between donors (Fig. 1C and D). As expected, pseudoislet size was highly consistent both within and between donors compared to intact islets. The coefficient of variation for cross‐sectional area and volume across all donors was reduced from 89% (mean ± SD =  0.039 ± 0.035 mm^2^) and 123% (mean ± SD =  0.057 ± 0.071 mm^3^) for cultured‐intact islets to 2.7% (mean ± SD = 0.034 ± 0.001 mm^2^) and 9.8% (mean ± SD = 0.034 ± 0.003 mm^3^) for pseudoislets. Average diameter of cultured islets from three donors varied from 141 ± 52 *μ*m to 306 ± 134, while that of pseudoislets were tightly distributed ranging from 199 ± 39 to 211 ± 9 *μ*m (all mean ± SD) among three donors (Fig. 1E).

**Figure 2:**
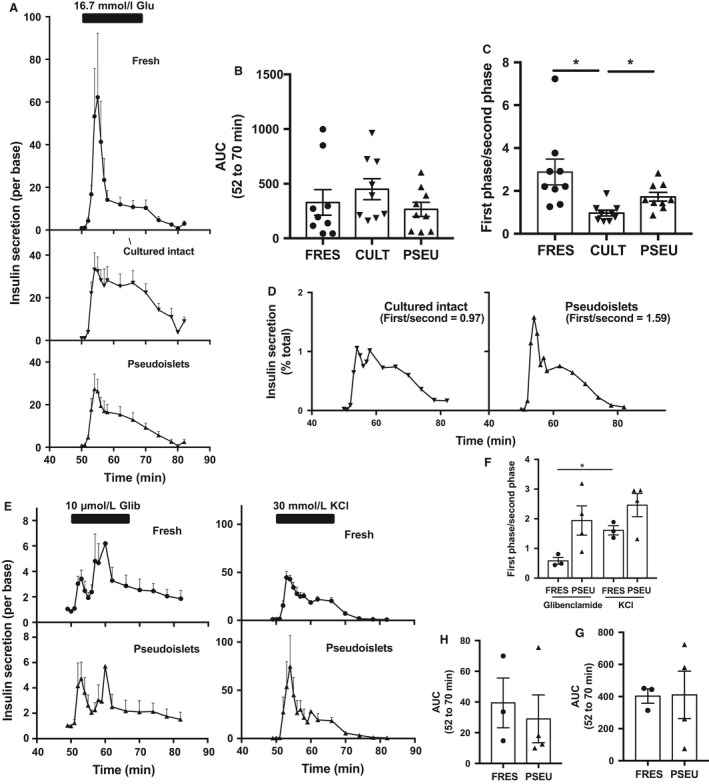
Insulin secretion of fresh, cultured‐intact, and pseudoislets in response to glucose, glibenclamide, and KCl in perifusion. (A) Perifusion profiles of fresh (FRES), cultured‐intact (CULT), and pseudo (PSEU) islets from nondiabetic donors in response to 16.7 mmol/L glucose (Glu) expressed taking basal insulin secretion as 1. Glucose ramp is indicated in a bar on the top. (B) Area under the curve (AUC) during glucose ramp in (A). (C) Ratio of stimulation index (SI) for the first phase over the second phase of insulin secretion in (A) determined as in methods. (D) Perifusion profiles of cultured intact and pseudoislets from donor 11. Mean ± SEM, *n* = 9 donors for all groups. (E) Perifusion profiles of FRES and PSEU in response to 10 *μ*mol/L glibenclamide (Gli) and 30 mmol/L KCl. Ramps with secretagogues are indicated in bars on the top.(F) Ratio of SI for the first phase over the second phase of insulin secretion in (E). AUC during ramps determined for (G) glibenclamide and (H) KCl. Mean ± SEM,* n* = 3 for fresh and 4 for pseudoislets. *P < 0.05.

Using a perifusion system, we compared GSIS of fresh islets with cultured‐intact and pseudoislets that were cultured for 1 week (Fig. 2A). GSIS expressed as area under the curve (AUC) was not different among the three groups (Fig. 2B). However, the sharp first‐phase insulin secretion seen in fresh human islets was markedly diminished in cultured‐intact islets (Fig. 2A). Although perifusion profiles of pseudoislets averaged from nine donors showed reduction in first phase compared with fresh islets, the first phase appears to be more prominent than that of cultured‐intact islets (Fig. 2A). Thus, we calculated the ratio of SI for first and second phase GSIS to express the prominence of first phase GSIS as we previously found that the ratio is a useful index when first‐phase insulin secretion was compared between human islets from nondiabetic and type 2 diabetic islets (Butcher et al. 2014). The ratio was reduced to 33.5% in cultured‐intact islets (mean ± SD = 0.97 ± 0.41) compared with fresh islets (mean ± SD = 2.88 ± 1.81, Fig. 2C, *P *< 0.05). Although pseudoislets showed a trend of reduction in first/second phase SI (mean ± SD = 1.73 ± 0.6) compared with fresh islets (*P* = 0.20), the ratio was higher compared with cultured‐intact islets (*P* < 0.05, Fig. 2C). To demonstrate how the ratio of first/second phase SI correlates with the prominence of first phase response, perifusion profiles of cultured‐intact and pseudoislets from donor 11 are shown in Figure 2D as this donor had first/second SI of 0.97 for cultured‐intact and 1.59 for pseudoislets, values representative of mean from nine donors. Next, we tested whether pseudoislets maintain secretory response to different secretagogues. Fresh islets increased insulin secretion to ~4 fold of baseline in response to 10 *μ*mol/L glibenclamide with gradual rise in second phase secretion and did not reduce insulin secretion swiftly after glibenclamide was removed (Fig. 2E). In comparison, 30 mmol/L KCl induced insulin secretion from fresh islets quickly and markedly to ~50 fold of baseline with wider first peak than glucose with swift return close to baseline after termination of KCl (Fig. 2E). These patterns were followed by pseudoislets (Fig. 2E), except there was a trend of higher first‐phase insulin secretion after glibenclamide exposure in pseudoislets compared with fresh islets (Fig. 2F, *P* = 0.068). Thus, the difference in first/second phase SI between glibenclamide and KCl was statistically significant only in a fresh islet group (Fig. 2F, *P* < 0.05). The magnitude of responses expressed as AUC did not differ between fresh islets and pseudoislets for both glibenclamide (Fig. 2G) and KCl (Fig. 2H).

### The heterogeneity of cell population in fresh islets is also seen in pseudoislets

**Figure 3:**
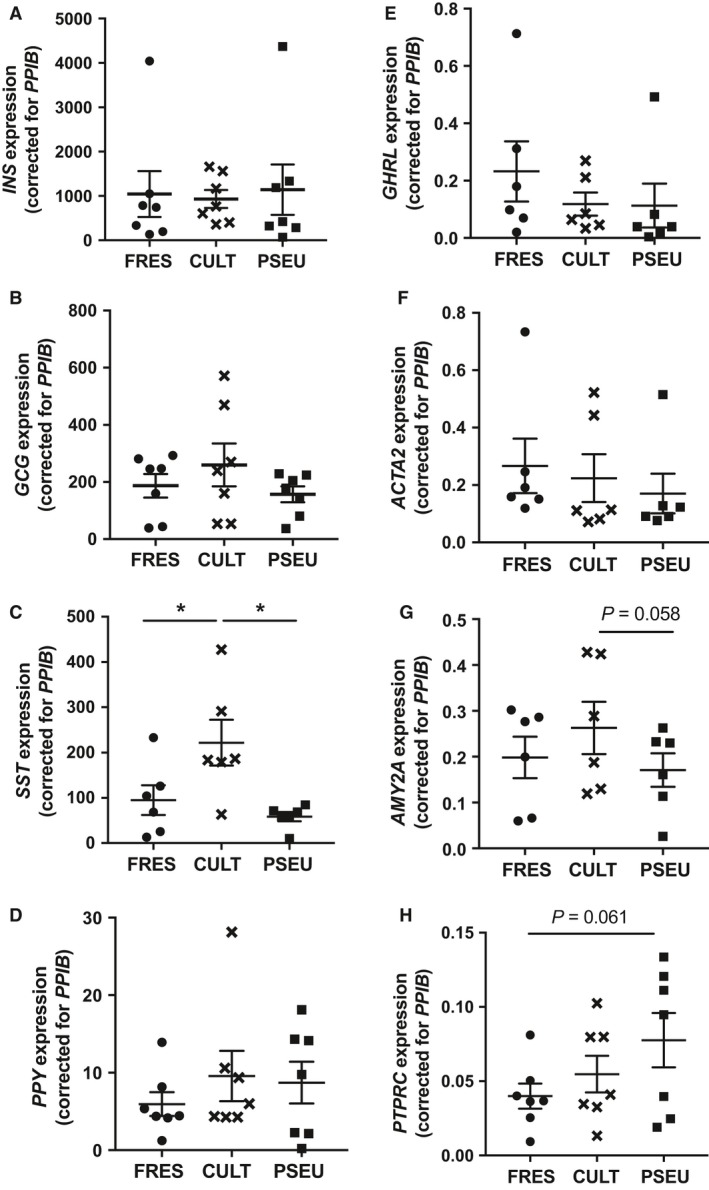
Expression of markers of islet cells in fresh, cultured‐intact, and pseudoislets. qPCR probed fresh (FRES), cultured‐intact (CULT), and pseudoislets (PSEU) for expression levels of cell type markers including (A) INS, (B) GCG, (C) SST, (D) PPY, (E) GHRL, (F) ACTA2 (G) AMY2A and (H) PTPRC. Expression of PPIB was used as internal control to correct expression values as in methods. Mean ± SEM. (A), (B), (D), and (H); *n* = 7 donors, (C), (E), (F), and (G); *n* = 6 donors. *P < 0.05.

Previously, immunostaining documented that pseudoislets contain insulin, glucagon, somatostatin, and pancreatic polypeptide positive cells (Zuellig et al. 2017; Yu et al. 2018) and indicated that the ratio of insulin/glucagon cells in the original intact islets and pseudoislets is similar (Zuellig et al. 2017). However, it is unknown whether minor population of nonendocrine cells in islets are incorporated into pseudoislets. Thus, we analyzed RNA expression of cell markers to detect a wide range of cell types including endocrine cells (*INS*,* GCG*,* SST*,* PPY*, and *GHR*), pancreatic stellate cells (PSC, *ACTA2*), acinar cells (*AMY2A*), and leukocytes (*PTPRC* aka CD45) (Fig. 3A–H). Cultured‐intact islets showed gene expression levels similar to fresh islets except for a 2.3‐fold increase in SST (Fig. 3C, *P* < 0.05). Pseudoislets showed a trend of increase in CD45 compared with fresh islets (*PTPRC*, Fig. 3H *P* = 0.061) and reduction in *AMY2A* compared with cultured‐intact islets (Fig. 3G, *P* = 0.058). However, all the other cell type markers seen in fresh islets were present at similar levels in pseudoislets indicating that dissociation and reaggregation do not preferentially select for certain cell types for inclusion in the pseudoislets.

### Pseudoislets show differential expression of extracellular matrix genes and inflammatory markers compared with fresh islets

**Figure 4:**
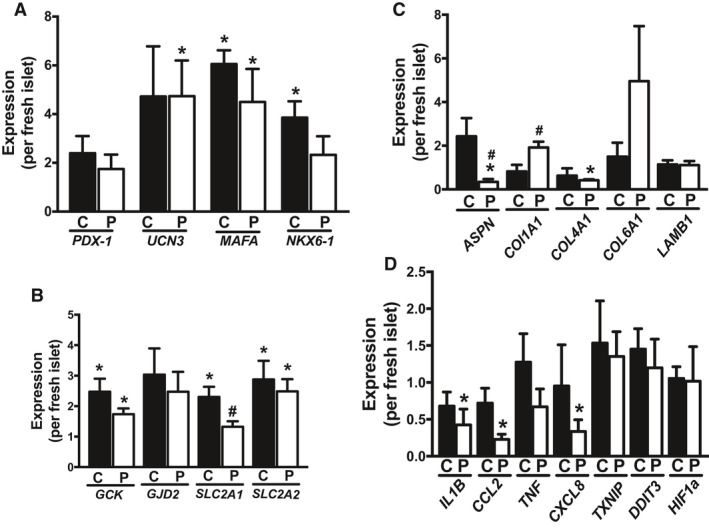
Expression of beta cell maturation, beta cell function, extracellular matrix, and stress markers in fresh, cultured‐intact, and pseudoislets. Expression of (A) markers associated with beta cell maturation, (B) beta cell function (C) extracellular matrix genes, and (D) stress associated genes determined by qPCR in cultured‐intact (C) and pseudo (P) islets taking an average for fresh islets as 1. Mean ± SEM. *n* = 5–11 donors (see Table 1 for assay performed for each donor). *P < 0.05 versus fresh islets. #; *P* < 0.05 versus cultured‐intact islets.

Next, we measured expression of beta cell maturation markers (Fig. 4A) and genes that support GSIS (Fig. 4B). Although beta cell maturation markers *UNC3* and *MAFA* (Romer and Sussel 2015; Liu and Hebrok 2017) were significantly increased in pseudoislets compared with fresh islets (Fig. 4A), *MAFA* and NKX6.1 were also increased in cultured‐intact islets compared with fresh islets. There was no difference between cultured‐intact and pseudoislets in expression of these genes (Fig. 4A). The expression of *GCK* and *SLC2A2* (GLUT2) was also increased in cultured‐intact and pseudoislets compared with fresh islets, while *SLC2A1* (GLUT1) was increased in cultured‐intact islets compared with fresh and pseudoislets (Fig. 4B). Overall, expression of these genes in pseudoislets is similar to cultured‐intact and does not explain difference in robustness of first phase GSIS among three groups of islets.

Since the loss of ECM during human islet isolation is considered to contribute to deterioration of insulin secretion and viability in cultured islets, ECM genes expressed in islets were analyzed (Roat et al. 2014; Llacua et al. 2018). In contrast to beta cell maturation markers and GSIS related genes that showed little difference between cultured‐intact and pseudoislets, there was differential expression of Asporin (*ASPN*) and type 1 collagen (*COL1A1*) between cultured‐intact and pseudoislets implicating changes in ECM in pseudoislets (Fig. 4C). Pseudoislets displayed elevated *COL1A1* compared with fresh islets (*P* < 0.05) and cultured‐intact islets (*P* < 0.05) and reduced expression of Asporin (*ASPN)* and type 4 collagen (*COL4A1*) compared with fresh islets (Fig. 4C, *P *< 0.05). We did not observe changes in type 6 collagen (*COL6A1*) or laminin (*LAMB1*). Considering that the loss of human islets during prolonged culture is associated with stresses including inflammation (Negi et al. 2012) and hypoxia (Komatsu et al. 2018), we tested genes known to be increased in beta cells under inflammation (*IL1B*,* CCL2,* and *CXCL8),* ER stress (*TXNIP* (Shalev 2014), *DDIT3*), and hypoxia (*HIF1A*). There was a significant reduction in expression levels of proinflammatory markers *IL1B*,* CCL2*, and *CXCL8* for pseudoislets compared with fresh islets (Fig. 4D, *P* < 0.05). However, pseudoislets did not show any difference in *TXNIP*,* DDIT3*, and *HIF1A* when compared with fresh or cultured‐intact islets. No significant change in levels of inflammatory and stress markers were observed for cultured‐intact islets compared with fresh islets (Fig. 4D).

### Pseudoislets can be efficiently transduced and maintain robust GSIS following lentiviral modification

**Figure 5:**
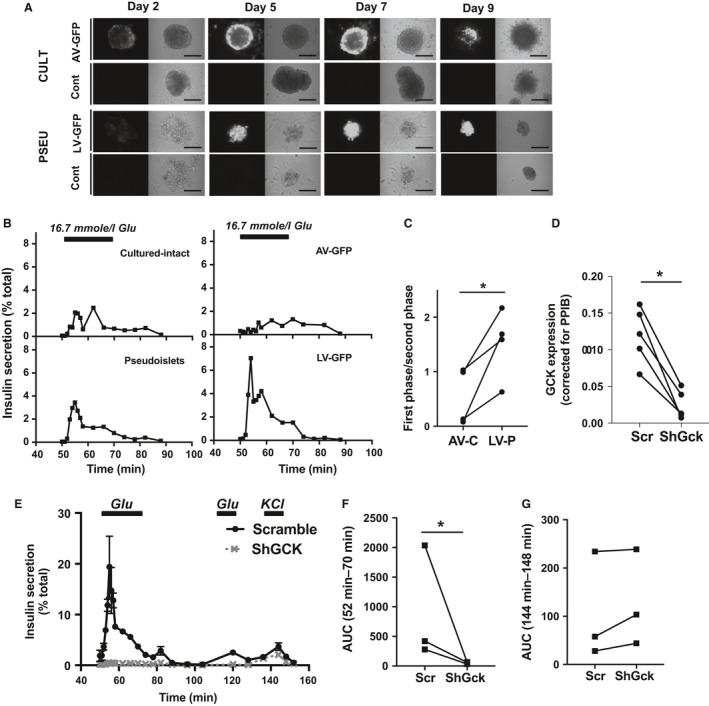
Lentivirus‐mediated transduction of pseudoislets. (A) Representative microscopy images of GFP fluorescence of AV‐CMV‐GFP (AV‐GFP) transduced cultured‐intact islets (CULT) and nontransduced control (Cont) as well as LV‐CMV‐GFP (LV‐GFP) transduced pseudoislets (PSEU) and nontransduced control (Cont) on Day 2, 4, 7, and 9 after transduction. Scale bar = 200 *μ*m. (B) Perifusion profiles of islets from (A). Glucose ramp (Glu) is indicated by the bar at the top. (C) Ratio of stimulation index for the first phase over the second phase of insulin secretion determined as in methods were compared between cultured‐intact islets treated with AV‐GFP (AV‐C) and pseudoislets treated with LV‐GFP/LV‐ShScramble (LV‐P) prepared from four donors. (D–G) Comparison of LV‐shScramble (Scr) and LV‐shGCK (ShGck) transduced pseudoislets. (d) qPCR determined *GCK* expression levels in Scr and ShGck pseudoislets and expressed using PPIB as internal control. Mean ± SEM. *n* = 5 donors. (E) Representative insulin secretion in response to 16.7 mmol/L glucose and 30 mmol/L KCl at indicated time in Scr and ShGck pseudoislets. Mean ± SEM of duplicates samples in donor 7 (Table 1). (F) Area under the curve (AUC) of insulin secretion during the first glucose ramp determined taking insulin secretion at base line as 1. (G) AUC of insulin secretion during KCl ramp determined taking insulin secretion at base line as 1. Mean ± SEM. *n* = 3 donors. *P < 0.05

The modulation of gene expression in intact human islets typically suffers low efficiency even when using adenovirus, which provides the best penetration into islets among viral vectors currently available. Thus, we compared the efficiency of transduction of human cultured‐intact islets using the *gold standard* adenoviral (AV) construct with pseudoislets modified during the dissociation phase using a lentiviral constructs (LV). As shown in Figure 5A, LV‐CMV‐GFP induced homogenous fluorescence throughout the entire pseudoislets rather than just the perimeter as was seen in AV transduction. The fluorescence signal induced by the AV‐CMV‐GFP in the cultured‐intact islets became gradually stronger as the culture duration was prolonged but remained weak to absent at the center of islet (Fig. 5A). Although GSIS pattern tested by perifusion did not differ between viral transduced and nontransduced islets, LV‐transduced pseudoislets (LV‐P) showed better preservation of first phase GSIS than AV‐transduced cultured‐intact (AV‐C) in agreement with Figure 2C and D (Fig. 5b). Better preservation of first phase was confirmed by the increase in SI ratio of first phase/second phase in LV‐P compared with AV‐C (Fig. 5C).

To demonstrate the utility of the pseudoislet platform as a tool to study the effect of gene downregulation on dynamism of GSIS, we performed LV‐mediated knockdown using shRNAs targeting GCK (shGck). GCK was targeted since genetic mutations in humans (Velho et al. 1992; Raimondo et al. 2014) and pharmacological inhibitions (Meglasson and Matschinsky 1986) of GCK are known to lead to a defect in GSIS, making it an appropriate positive control. Transduction with LV‐shGck resulted in significant downregulation of the *GCK* gene in comparison with LV‐shScramble (ShScr) transduced pseudoislets when measured by qPCR (Fig. 5D, *P* < 0.05). In addition, both the first and second phase of GSIS was completely lost in LV‐shGck infected pseudoislets while the LV‐shScr treated pseudoislets had preserved first and second phase GSIS (Fig. 5E and F) similar to pseudoislets without viral infection (Fig. 2A and D). Despite the loss of glucose responsiveness, 30 mmol/L KCl induced insulin secretion in pseudoislets transduced by either LV‐shGck or LV‐shScramble constructs (Fig. 5E and G). This is in line with GCK's role in glycolysis upstream of closure of K_ATP_ channel in beta cells (Lenzen 2014), and demonstrates the LV transduction blocked glucose responsiveness specifically without impacting exocytosis machinery

## Discussion

Results from the current study demonstrate that human pseudoislets can serve as a valuable tool to study the dynamic regulation of insulin secretion using gene downregulation techniques. Side by side comparison of AV‐transduced intact islets and LV‐P showed that LV‐P have a better preserved first phase of GSIS compared with AV‐C. We chose GCK, MODY2 gene that is known to impair GSIS (Velho et al. 1992; Raimondo et al. 2014), to validate efficiency of gene downregulation in human pseudoislets by lentivirus both at gene expression level and by GSIS. LV‐shGck reduced *GCK* expression significantly and caused profound defects in first and second phase GSIS while preserving KCl response in human pseudoislets providing a proof of principle for assessment of both phases of insulin secretion in genetically modified human pseudoislets. Functional and molecular characterizations of the pseudoislets reveal a high similarity in gene expression profile of cell type markers and response to secretagogues between human pseudoislets and the original fresh islets. Thus, human pseudoislets created by a simple protocol serve as a useful tool to assess the genetic control of the dynamic regulation of insulin secretion within three‐dimensional structures retaining nonbeta cell neighbors. Lentiviral‐mediated *over*‐expression of genes such as connexins and SIX3 previously showed utility of lentivirus‐transduced human pseudoislets in assessment of GSIS (Caton et al. 2003; Arda et al. 2016). Recent publication of lenti‐shRNA mediated downregulation of BCL11A (Peiris et al. 2018) and our current study targeting GCK provide strong support that pseudoislets are effective in the assessment of functional gene *down*regulation, which requires high efficiency. Moreover, our data indicate that this platform enables future studies to understand the contribution of genes of interest in a model with better preserved first and second phase GSIS.

First‐phase GSIS in humans is considered to play a critical role in the prevention of postprandial hyperglycemia and is known to be lost in diabetes at the early stage of the disease (Shichiri et al. 2000; Bonora et al. 2006; Monnier et al. 2007). Blunting of first‐phase insulin secretion in perifusion was seen when human islets isolated from diabetic donors were compared with those from nondiabetic donors indicating that the prominence of first phase represented by first/second phase ratio is an important parameter of human islet function (Butcher et al. 2014). Critically, restoration of first phase could potentially impact glycemic control in humans. Since the first‐phase insulin secretion is more prominent in human islets than in mouse islets, testing in human islets is essential to understand the mechanisms regulating first‐phase insulin secretion (Arrojo e Drigo et al. 2015). However, dispersion of human islets to introduce or knockdown transgenes or prolonged culture of intact islets blunts first‐phase insulin secretion. Thus, preservation of first‐phase GSIS in pseudoislets offers an advantage over both dispersed and cultured‐intact islets. While our current study focused on dynamism of GSIS, Yu et al. demonstrated that human pseudoislets made by controlled aggregation of dispersed islet cells, an approach employed by us as well, improve insulin secretion corrected by cell number highlighting the advantage of pseudoislets over dispersed islet cells for the efficiency of insulin secretion (Yu et al. 2018). Secretory profile of human pseudoislets to KCl and glibenclamide was also similar to that of fresh islets further supporting the utility of human pseudoislets to dissect the regulation of insulin secretion in human islets.

To avoid biasing our gene expression and insulin secretion data by using pseudoislets with significantly reduced size compared with fresh and cultured‐intact islets, we created pseudoislets with 3000 islet cells and obtained pseudoislets of ~200 *μ*m. Further improvement in first‐phase GSIS of pseudoislets could be possible by reducing size of pseudoislets. Zuellig et al. (2017) used 250–1000 cells per pseudoislets and observed inverse correlation between GSIS and size of pseudoislets. Also, methods to form pseudoislets, such as hanging drop (Zuellig et al. 2017), a low attachment well (the current study, (Arda et al. 2016)), and agarose microwell (Hilderink et al. 2015), may affect function of pseudoislets. Although perifusion was not preformed, a bioengineered microwell that recently became commercially available was reported to allow the formation of human pseudoislets with robust GSIS using a similar simple step as ours (Yu et al. 2018) and may further improve first‐phase GSIS compared with a low attachment well we used in the current study.

All endocrine cell types, acinar cell, leukocyte, and activated PSC markers were expressed in our pseudoislets, all at very similar levels as fresh islets. Combined with previous histological studies that demonstrated similarity in endocrine cell composition and distribution (Zuellig et al. 2017; Yu et al. 2018), human pseudoislets mirror the original intact islets well for both nonendocrine and endocrine cells. When sorted mouse beta, alpha, and delta cells were used, alpha and delta cells were incapable to form aggregates implicating beta cells are critical for the formation of pseudoislets (Reissaus and Piston 2017). Although it remains to be determined whether beta cell drives formation of pseudoislets in humans, our data indicate that the formation of pseudoislets have little selectivity in inclusion or exclusion of cell types to be incorporated for both endocrine and nonendocrine cells.

While higher expression of beta cell maturation markers (*UNC3*,* MAFA*) and beta cell function markers (*GCK*, and *SLC2A2)* in pseudoislets compared with fresh islets implicate good maintenance of beta cell identity in pseudoislets, we did not detect difference in these markers between cultured‐intact and pseudoislets. Thus, the robust first‐phase GSIS in pseudoislets compared with cultured‐intact islets cannot be explained by better preservation of differentiation status in pseudoislets. Interestingly, we observed that *COL1A1* expression was increased while *ASPN* expression was reduced in pseudoislets compared with cultured‐intact islets indicating remodeling of ECM unique to pseudoislets. The loss of ECM during islet isolation is considered to impair viability and function of human islets (Arous and Wehrle‐Haller 2017). The provision of ECM components either as peptides, acellular matrix, or mesenchymal stem cells improves functional mass of cultured human islets supporting the importance of ECM in islet health (Arrojo e Drigo et al. 2015; Arzouni et al. 2018). While the implication of change in expression of these genes on the property of ECM in pseudoislets remains to be determined, ECM remodeling may aid pseudoislets to maintain insulin secretion over long‐term culture. ASPN, a small leucine‐rich proteoglycan expressed highly in activated PSC, activates NF‐*κ*B and promotes endothelial mesenchymal transition in pancreatic cancer (Wang et al. 2017). While its action on pancreatic islets is unknown, *Aspn* was one of differentially expressed genes in microarray analysis of mouse islets after high fat diet (Imai et al. 2008; Roat et al. 2014). Combined with the reduced expression of proinflammatory cytokines (*IL1B*,* CCL2,* and *CXCL8*) in pseudoislets compared with fresh islets, ECM remodeling and reduced ASPN‐mediated inflammation may contribute to preserve beta cell function in pseudoislets. As for cultured intact islets, SST expression was twofolds higher than both fresh and pseudoislets. If the increase in SST expression is associated with the elevation of SST secretion in cultured‐intact islets, the inhibition of GSIS by SST might contribute to reduced first phase GSIS in intact islets.

Well‐maintained expression of beta cell maturation markers and genes associated with beta cell function in cultured‐intact islets was surprising considering that a previous microarray study reported reduction in *MAFA, NEUROD1,* and other transcription factors associated with maturation when human islets cultured for 3 days were compared with fresh islets captured by laser microdissection (Negi et al. 2012). This could be due to selection bias so that the culture‐intact islet group is enriched with a population of cells that survived culture (Paraskevas et al. 2000; Arzouni et al. 2017). There exists functional heterogeneity among beta cells, some showing more maturity and higher functionality than others (Liu and Hebrok 2017; Benninger and Hodson 2018). It will be intriguing to determine whether more mature beta cells are preferentially surviving during culture of human islets. Also, the fresh islets we used were cultured for 1–2 days prior to RNA extraction, which might downregulate maturation markers compared with islets obtained by laser microdissection used in the previous study (Negi et al. 2012).

Our study has a couple of limitations. Although qPCR allowed the demonstration of wide population of cell markers including low abundant cells, flow cytometry or single cell sequencing is needed to obtain accurate composition of intact‐ and pseudoislets. Similarly, alterations of ECM and proinflammatory cytokine gene expression observed by qPCR need confirmation at protein levels before their implication for the preservation of function of pseudoislets is established. As we did not perform unbiased gene expression profiling, there could be additional differentially regulated genes in pseudoislets compared with fresh and cultured‐intact islets that have yet to be revealed.

In summary, we demonstrated that human pseudoislets created using a simple protocol maintain functional and molecular characteristics of the original islets, preserve robust first‐phase GSIS after prolonged culture, and serve as an efficient gene transduction platform to test gene function in human islets in culture.

## Conflict of Interest

None of the authors have conflicts of interest related to this publication.
